# Inactivated vaccines derived from bovine viral diarrhea virus B3 strain elicit robust and specific humoral and cellular immune responses

**DOI:** 10.3389/fmicb.2025.1607334

**Published:** 2025-08-08

**Authors:** Ming-Guo Xu, Ao-Di Wu, Jing-Nan Liu, Zhong-Lian Yang, Hong-Huan Li, Hai-Long Ma, Ning-Ning Yang, Jin-Liang Sheng, Chuang-Fu Chen

**Affiliations:** ^1^College of Animal Science and Technology, Shihezi University, Shihezi, China; ^2^Changji Hui Autonomous Prefecture Animal Disease Prevention and Control Center, Changji, China; ^3^Department of Biotechnology, Linxia Modern Career Academy, Linxia, China; ^4^College of Animal Science and Technology, Xinyang Agriculture and Forestry University, Xinyang, China

**Keywords:** BVDV, B3, Xinjiang, inactivated vaccines, immune responses

## Abstract

**Introduction:**

Bovine viral diarrhea-mucosal disease (BVD-MD) is a significant viral disease in cattle caused by infection with bovine viral diarrhea virus (BVDV). Xinjiang, a major pastoral region in China, is heavily affected by this disease. Owing to the high genetic variability of BVDV, developing cross-protection vaccines targeting predominant strains is essential.

**Methods:**

In this study, inactivated vaccines were developed using BVDV strains isolated from Xinjiang, including BVDV-1 (B1), BVDV-2 (B2), XJ-BVDV-3 (B3), and BVDV-LC (LC). BALB/c mice were immunized, and immune responses were assessed via ELISpot, ELISA, and virus neutralization assays. On day 42 post-immunization, the mice were challenged with BVDV, and the viral loads were quantified.

**Results:**

The vaccines showed stable characteristics and sterility and are suitable for immunization studies. All the vaccines stimulated interferon-gamma (IFN-γ) production, and the IFN-γ levels in the B1 and B3 groups were significantly higher than those in the commercial vaccine group (*p* < 0.0001). The ELISA results revealed specific IgG, IgG1, and IgG2a antibodies. Neutralization assays revealed that the B3- and LC-inactivated vaccine groups presented significantly higher neutralizing antibody titers than did the commercial vaccine group ((*p* < 0.05). Tissue viral load detection revealed that the inactivated vaccines reduced the viral load across various tissues.

**Discussion:**

In conclusion, this study developed inactivated vaccines from the B1, B2, B3, and LC strains, all of which induce robust immune responses. Among these, the B3 inactivated vaccine show great potential for commercialization, providing a valuable reference for BVDV prevention and control in Xinjiang.

## Introduction

Bovine viral diarrhea-mucosal disease (BVD-MD) is a significant viral infection caused by bovine viral diarrhea virus (BVDV), which represents a major threat to the global cattle industry (Shirai et al., 1984; Shimizu et al., 1989). The virus was first identified by Olafson (1946) in cattle exhibiting symptoms such as digestive ulcers and diarrhea in New York. In 1957, the virus was successfully isolated (Lee and Gillespie, 1957). In China, BVDV was first isolated in Changchun, Jilin Province, in 1980, and subsequent studies confirmed that BVDV infection was widespread among domestic cattle populations (Li et al., 1983). BVDV is prevalent in most regions of China, with a particularly high incidence in pastoral areas where live-stock farming is well developed.

BVDV is a single-stranded, positive-sense RNA virus with a genome size of approximately 12.3–13 kb encoding approximately 3,988 amino acids (Simmonds et al., 2017). The genome consists of a single open reading frame (ORF), flanked by 5′- and 3′-untranslated regions (5′-UTRs and 3′-UTRs), which form critical secondary structures essential for genome replication and translation (Deng and Brock, 1992; Simmonds et al., 2017). The ORF encodes a large polyprotein with an estimated molecular weight of 449 kDa, which is cleaved by both viral and host proteases to produce four structural proteins (C, E0/Erns, E1 and E2) and eight nonstructural proteins (Npro, p7, NS2, NS3, NS4A, NS4B, NS5A, and NS5B) (Collett et al., 1988; Deng and Brock, 1992; Al-Kubati et al., 2021).

As a prominent member of the *Pestivirus* genus within the *Flaviviridae* family, BVDV is closely related to classical swine fever virus (CSFV) and border disease virus (BDV), which are also classified within the same genus (Simmonds et al., 2017). According to recent updates from the International Committee on Taxonomy of Viruses (ICTV), the number of species within the *Pestivirus* genus has expanded from four to seven (Simmonds et al., 2017). BVDV is classified into three genotypes: BVDV-1 (*Pestivirus* A), BVDV-2 (*Pestivirus* B), and BVDV-3 (*Pestivirus* H), with BVDV-3 also known as HoBi-like *Pestivirus* or atypical *Pestivirus* (Simmonds et al., 2017). Genotype BVDV-1 includes at least 23 subtypes (1a–1w), whereas BVDV-2 consists of four subtypes (2a–2d). Furthermore, BVDV-3 is geographically diverse, with strains identified in regions such as Brazil, Thailand, and Italy (Yeşilbağ et al., 2017; Deng et al., 2020). Among these, BVDV-1 is the most widespread genotype globally and is commonly used in laboratory research and vaccine development. In contrast, BVDV-2 is more pathogenic and is as-associated with acute infections and severe clinical manifestations. BVDV can be further categorized into two biotypes, cytopathic (cp) and noncytopathic (ncp), on the basis of their ability to induce a cytopathic effect (CPE) in cell cultures. While most *pestiviruses* are ncp, both cp and ncp variants have been identified across the three genotypes.

BVDV is widely distributed and has a complex pathogenic mechanism, including the capacity to cause immunosuppression in animals. Currently, no specific therapeutic treatments exist for BVDV. As a result, vaccination and culling of persistently infected animals remain the primary strategies for their control and eradication. Several European countries have initiated national BVDV eradication programs; however, similar efforts have not yet been implemented in China (Moennig et al., 2005; Graham et al., 2015; Wernike et al., 2017). This gap has driven ongoing efforts to develop safer and more effective vaccines to improve BVDV control. Coggins et al. (1961) first reported a modified live virus (MLV) BVDV vaccine. Since then, both MLV and inactivated vaccines have been developed. Owing to safety concerns, inactivated vaccines offer distinct advantages over MLV vaccines. These vaccines are produced by chemical, thermal, or radiation treatment to render the pathogen nonviable while still eliciting a robust immune response. Inactivated vaccines are widely regarded as safe and effective means of disease prevention and play a pivotal role in controlling the spread of infectious diseases. Chinese researchers, including Hou et al. (2006) successfully developed an inactivated BVDV vaccine in 2006 via a virus-like particle oil emulsion adjuvant. The vaccine was shown to rapidly induce antibody production, sustain long-term immunity, and provide strong protection. Additionally Wang et al. (2014) developed an inactivated vaccine using the BVDV-1a (NM01) strain, which demonstrated cross-protection against BVDV-1b infections. The BVDV-1 NM01 strain has since become one of the predominant antigen strains used in vaccines in China.

The development of vaccines against BVDV in China is still in its early stages, with only three inactivated vaccines based on BVDV-1 strains currently approved for market release, which is insufficient to meet the growing demand. Owing to the high genetic diversity of BVDV, the successful development of an effective inactivated vaccine requires careful selection of suitable virus strains, adoption of efficient inactivation methods, and implementation of rigorous quality control and immunological evaluation. These factors are critical to ensuring the safety and efficacy of the vaccine. In this study, we selected four BVDV strains isolated from Xinjiang and characterized in our laboratory: BVDV-1 (B1), BVDV-2 (B2), XJ-BVDV-3 (B3), and BVDV-LC (LC). These strains were inactivated with 3% hydrogen peroxide (H_2_O_2_) and emulsified with Freund’s adjuvant to increase immunogenicity, resulting in inactivated vaccines. The immunogenicity of the resulting vaccines was evaluated by immunizing BALB/c mice, providing scientific evidence supporting the development of effective BVDV control strategies in Xinjiang.

## Materials and methods

### Experimental mice

Ninety female BALB/c mice, aged 6–8 weeks and with a mean body weight of 20 ± 5 g, were purchased from the Animal Experiment Center of Xinjiang Medical University. The mice were provided with access to adequate food and water and maintained on a 12 h light–dark cycle at 15–20°C and 50% relative humidity.

### Cell and virus culture

Madin–Darby bovine kidney (MDBK) cells were obtained from the National Collection of Authenticated Cell Lines (Shanghai, China). These cells were cultured in Dulbecco’s modified Eagle’s medium (DMEM; Gibco, United States) supplemented with 10% fetal bovine serum (FBS; Gibco, United States) and incubated at 37°C in a 5% CO_2_ atmosphere to promote growth. The BVDV strains used included B1, B2, B3, and LC, all of which were identified and maintained by the Zoonotic Diseases Laboratory at Shihezi University. These strains were propagated in MDBK cells, and the 50% tissue culture infective dose (TCID_50_) was determined via the Reed–Muench method (Reed and Muench, 1938).

### Virus inactivation and detection

After reviewing the product information for commercial BVDV-inactivated vaccines, we found that the viral antigen content was at least 10^7^ TCID_50_/mL (Wu et al., 2017). Therefore, we standardized the viral antigen content of our inactivated vaccine to 10^7.5^ TCID_50_/mL (Wang et al., 2019). For strain inactivation as previously reported, 3% H_2_O_2_ (FUYU CHEMICAL, China) was added to the viral solutions of B1, B2, B3, and LC, and the mixtures were then inactivated in a water bath at 27°C for 2 h (Li et al., 2024). After inactivation, the viruses were inoculated into MDBK cells and passaged for five generations via our established indirect immunofluorescence assay (IFA) and reverse transcription-polymerase chain reaction (RT-PCR) methods to assess inactivation efficacy (Yang et al., 2023). If the inactivated B1, B2, B3, and LC strains show no green fluorescence in MDBK cells during IFA and yield negative RT-PCR results, complete virus inactivation can be confirmed.

### Vaccine preparation using inactivated viruses

Antigen quantification was performed using IFA to ensure accurate determination of the antigen content in each vaccine formulation. Inactivated B1, B2, B3, and LC virus antigens (10^7.5^ TCID_50_/mL) were combined with Freund’s complete or incomplete oil-in-water adjuvant (Sigma-Aldrich, United States) in a 1:1 volume ratio. The resulting mixture was emulsified using an emulsifier (FLUKO, China). As described in previous studies (Liu, 2017; Wang, 2013), the prepared inactivated vaccines underwent a series of tests, including visual inspection, formulation analysis, viscosity measurement, stability assessment, and sterility testing.

### Experimental design using mice

**Figure 1:**
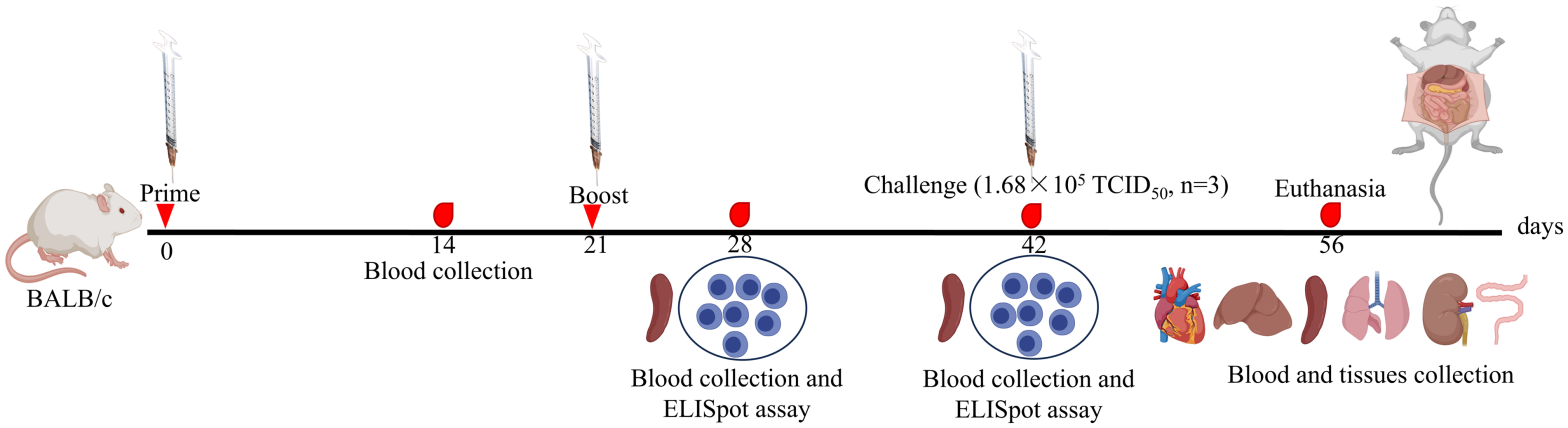
Schematic diagram of the experimental design and sample collection.

Ninety female BALB/c mice were randomly assigned to six groups of 15 for a 56-day study. The mice received subcutaneous (s.c.) vaccinations on days 0 and 21, with the phosphate-buffered saline (PBS; Biosharp, China) immunization group serving as the negative control (NC) and the commercial vaccine (TECON, China) group serving as the positive control (PC; Table 1). After vaccination, adverse effects were monitored in real time, and blood samples were collected at designated time points (Figure 1). Serum samples were separated and temporarily stored at −20°C.

**Table 1:** Experimental grouping.

Groups (*n* = 15)	Immunogen and dosage (μL)	Immunization time (*d*)
PBS (negative control, NC)	200	0, 21th
B1 + Freund’s adjuvant	200	0, 21th
B2 + Freund’s adjuvant	200	0, 21th
B3 + Freund’s adjuvant	200	0, 21th
LC + Freund’s adjuvant	200	0, 21th
Commercial vaccine (positive control, PC)	100	0, 21th

### Preparation of mouse spleen lymphocytes

On days 28 and 42 post-immunization, three mice (*n* = 3) from each group were randomly selected and euthanized by cervical dislocation. The mice were then disinfected by immersion in 75% ethanol for 20 min. Spleen lymphocytes were isolated aseptically according to the manufacturer’s protocol (TBD, China). Briefly, the spleen was excised and placed in a 2 mL centrifuge tube containing 1 mL of homogenization wash solution, after which the tissue was minced. The minced tissue was filtered through a 70 μm sieve (Biosharp, China) and gently ground to create a cell suspension. This suspension was centrifuged at 400 × g for 10 min, and the supernatant was discarded. The pellet was resuspended in 1 mL of dilution buffer. Next, in a separate 15 mL centrifuge tube, 5 mL of lymphocyte separation solution was added, and the resuspended cells were carefully layered on top. The mixture was subsequently centrifuged at 600 × g for 30 min to isolate the lymphocyte layer. The resulting milky layer was collected, mixed with 5 mL of wash solution, and centrifuged again at 400 × g for 10 min. After the supernatant was removed, the cell pellet was resuspended in RPMI-1640 medium (Gibco, United States) supplemented with 10% FBS. Finally, cell viability was assessed via 0.4% trypan blue (TBD, China) staining, followed by cell counting (Nexcelom Bioscience, United States).

### Enzyme-linked immunospot assay

The enzyme-linked immunospot (ELISpot) assay is considered one of the most reliable methods for detecting antigen-specific T cells in both mice and humans (Slota et al., 2011). Therefore, a mouse interferon-gamma (IFN-γ) ELISpot assay was performed to assess cellular immune responses. Briefly, mouse lymphocytes (1 × 10^6^ cells/well) were seeded into a 96-well ELISpot plate precoated with an anti-IFN-γ antibody (Mabtech, Sweden). The experimental groups were stimulated with the corresponding antigen (10 μg), with concanavalin A (ConA; 10 μg; Biosharp, China) serving as the PC and PBS as the NC. The plate was incubated at 37°C in a 5% CO_2_ atmosphere for 30 h. Following incubation, IFN-γ spot-forming cells (SFCs) were detected according to the manufacturer’s instructions (Mabtech, Sweden).

### Determination of BVDV-specific antibodies

An indirect enzyme-linked immunosorbent assay (ELISA) was used to detect specific antibodies, including IgG, IgG1, and IgG2a, in mouse serum. B1, B2, B3, and LC whole viruses were diluted according to preset conditions and added to a 96-well ELISA plate at 100 μL per well. The plate was incubated overnight at 4°C for antigen coating. After the coating solution was removed, the wells were washed twice with PBS containing 0.05% Tween-20 (PBST; Solarbio, China) and then dried. Next, each well was blocked by adding 200 μL of 5% nonfat dry milk (BD, United States) and incubated at 37°C for 2 h to block nonspecific binding. The wells were subsequently washed twice with PBST and dried again. Diluted serum samples (100 μL per well) were added and incubated at 37°C for 1 h. After five additional washes with PBST, 100 μL of HRP-conjugated goat anti-mouse IgG, IgG1, or IgG2a was added, and the mixture was incubated at 37°C for 1 h. After five more washes with PBST, 100 μL of tetramethylbenzidine (TMB) substrate (Solarbio, China) was added, and the reaction was allowed to develop for 15 min at 37°C in the dark. The reaction was terminated by the addition of 50 μL of ELISA stop solution (Solarbio, China), and the optical density (OD) at 450 nm was measured via a microplate reader (TECAN, Switzerland).

### Determination of neutralizing antibodies against the B1 strain

The virus neutralization test (VNT) is considered the gold standard for the serological diagnosis of BVDV and CSFV infections and is also used as a reference efficacy test for commercial vaccines (Chimeno Zoth et al., 2007; Yi et al., 2022). Previous studies have shown that the BVDV-1 strain has the broadest geographic and temporal distribution; thus, we selected the B1 strain for the VNT (Zhang et al., 2022; Zhu et al., 2022). Serum samples were heat-inactivated by incubation in a water bath at 56°C for 30 min. Following inactivation, the serum was serially diluted twofold, ranging from 1:2 to 1:256, in DMEM. Equal volumes of the B1 virus (100 TCID_50_) and diluted serum were then mixed and incubated with 80% confluent MDBK cells for 2 h at 37°C. After incubation, the virus-serum mixture was discarded, and the cells were cultured in growth medium supplemented with 1% FBS for 5–7 days at 37°C in a 5% CO_2_ atmosphere. CPE was assessed visually under an inverted microscope (Nikon, Japan), and the 50% neutralizing antibody titer was calculated via the Reed–Muench method (Reed and Muench, 1938).

### Viral challenge experiment

Previous studies have demonstrated that BVDV-1 strains present the highest incidence rates in both swine and cattle populations (Deng et al., 2012; Zhang et al., 2022). Consequently, we selected the B1 strain for a murine challenge model. The challenge dose and administration route were based on our earlier findings, in which each mouse received an intraperitoneal injection of 1.68 × 10^5^ TCID_50_ of the virus on day 42 post-initial immunization (Yang et al., 2022).

### Viral load quantification

The primer pair was designed to amplify a 196 bp fragment of the 5′-UTR of the BVDV genome, which served both to construct a standard vector and to quantify viral RNA. The forward primer sequence was 5′-GTAGTCGTCAGTGGTTCG-3′, and the reverse primer sequence was 5′-GCCATGTACAGCAGAGAT-3′. For standard vector construction, the purified RT-PCR product was cloned and inserted into the pMD19-T vector (TaKaRa, Japan) according to the manufacturer’s protocol. Plasmid copy number was determined via the method outlined by Sivaganesan et al. (2008). A standard curve was generated by plotting the Ct values of serial 10-fold dilutions of the plasmid, which were run alongside the test cDNA samples.

On day 14 post-challenge, blood samples were collected via retro-orbital venipuncture via a capillary tube and transferred into anticoagulant-treated tubes. The mice were euthanized by cervical dislocation, and the heart, liver, spleen, lung, kidney, and small intestine were carefully harvested. Total RNA was extracted from each tissue sample following a standardized protocol and reverse transcribed into cDNA. The viral load in each tissue sample was quantified via reverse transcription quantitative PCR (RT-qPCR).

### Statistical analysis

GraphPad Prism 8.0 (GraphPad Software, Inc., United States) was used for statistical analysis and graphical representation. One-way or two-way analysis of variance (ANOVA) was conducted according to the experimental design, and statistical significance was considered at *p* < 0.05. Microsoft Excel was used to generate standard curves, calculate the TCID_50_, and quantify the viral loads in the tissues. All the experiments were conducted in triplicate. The data are presented as the means ± standard deviations (SDs).

## Results

### H_2_O_2_ inactivated BVDV

IFA analysis revealed that the inactivated B1, B2, B3, and LC strains did not induce green fluorescence in MDBK cells, confirming their non-infectivity. Furthermore, RT-PCR failed to amplify specific viral bands, further confirming the complete inactivation of the viruses (Supplementary Figure 1).

### Preparation of inactivated vaccines

We developed four inactivated vaccines, B1, B2, B3, and LC, each of which was formulated with Freund’s adjuvant. A series of evaluations demonstrated that these vaccines meet the requirements for subsequent preclinical studies (Supplementary Figure 2).

### The effects of inactivated vaccines on cytokine expression

**Figure 2:**
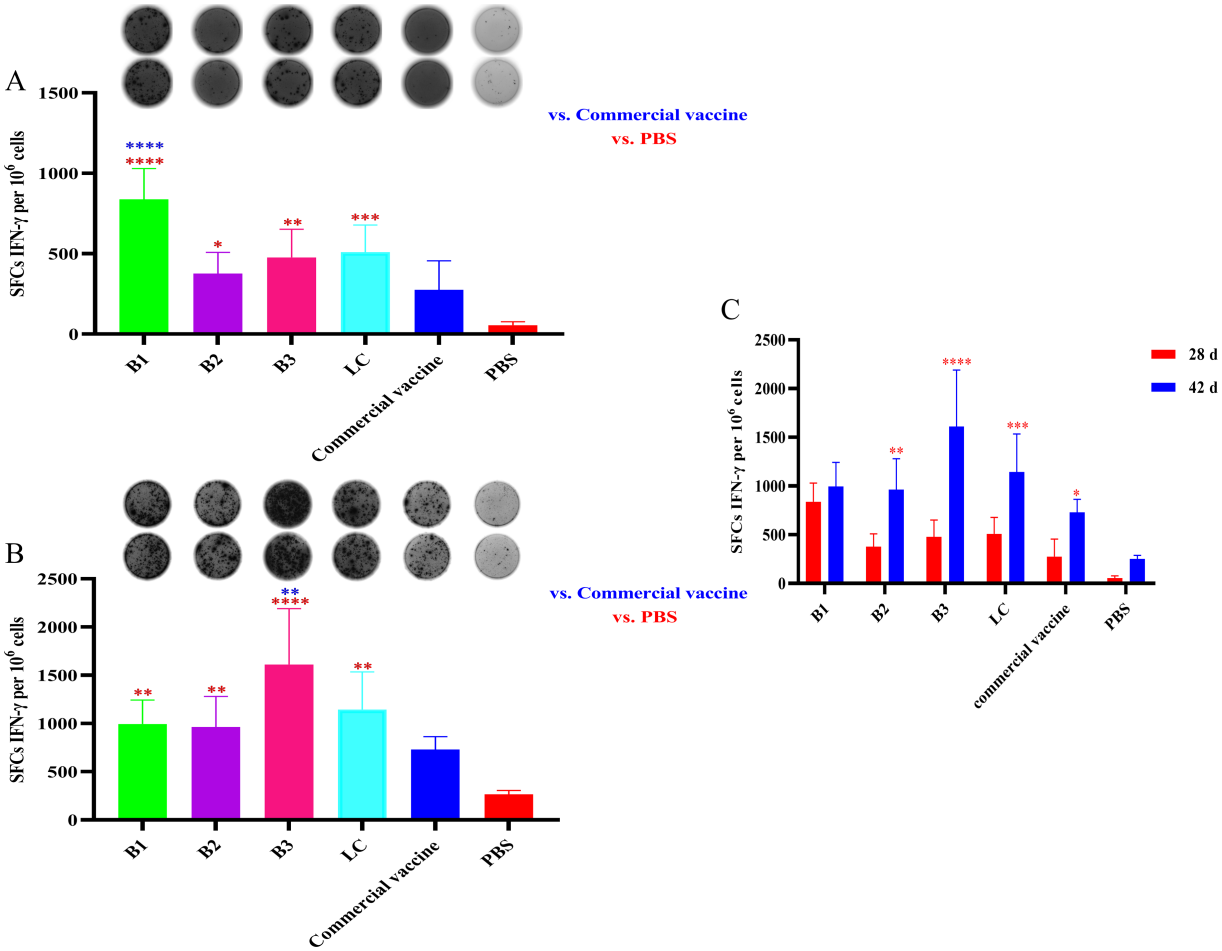
IFN-γ secretion in spleen lymphocytes of mice vaccinated with inactivated vaccines. The mice were immunized with in-house-prepared inactivated vaccines, with a commercial vaccine and PBS used as PC and NC, respectively. Splenocytes were harvested on days 28 and 42 post-immunization and restimulated with the corresponding antigen used in vaccination. An ELISpot assay was used to quantify the number of IFN-γ-secreting SFCs per 1 × 10^6^ splenocytes. **(A)** Spot diagram and statistical analysis of IFN-γ-secreting spots in mice immunized for 28 days. **(B)** Spot diagram and statistical analysis of IFN-γ-secreting spots in mice immunized for 42 days. **(C)** Comparison of IFN-γ-positive spots between mice immunized for 28 and 42 days. Statistical significance was assessed by one-way or two-way tests, with ^*^p < 0.05, ^**^p < 0.01, ^***^p < 0.001, and ^****^p < 0.0001. Blue indicates the comparison of in-house-prepared inactivated vaccines and NC group vs. the PC group; red indicates the comparison of in-house-prepared inactivated vaccines and PC group vs. the NC group.

The number of IFN-γ-secreting SFCs was measured in 1 × 10^6^ splenocytes from immunized mice via the ELISpot assay. As shown in Figure 2A, at 28 days post-immunization, splenocytes from mice immunized with the inactivated vaccines B1, B2, B3, or LC presented significantly more IFN-γ-positive spots than those from the NC group did (*p* < 0.05). Notably, the B1 group presented markedly higher numbers of IFN-γ-positive spots than did the PC group (*p* < 0.0001). At 42 days post-immunization, splenocytes from the B1, B2, B3, and LC groups continued to display significantly more IFN-γ-positive spots than those from the NC group did (Figure 2B; *p* < 0.01). Moreover, the B3 vaccine group presented significantly higher numbers of IFN-γ-positive spots than did the PC group (*p* < 0.0001). These findings suggest that immunization with in-house prepared inactivated vaccines effectively stimulates IFN-γ production in mouse splenocytes. Additionally, IFN-γ secretion increased over time, with significant increases observed in the B2, B3, LC, and PC groups (Figure 2C; *p* < 0.05). Overall, these data indicate that in-house-prepared inactivated vaccines may have advantages in stimulating IFN-γ production compared with NC.

### BVDV-specific IgG, IgG1, and IgG2a antibody levels

**Figure 3:**
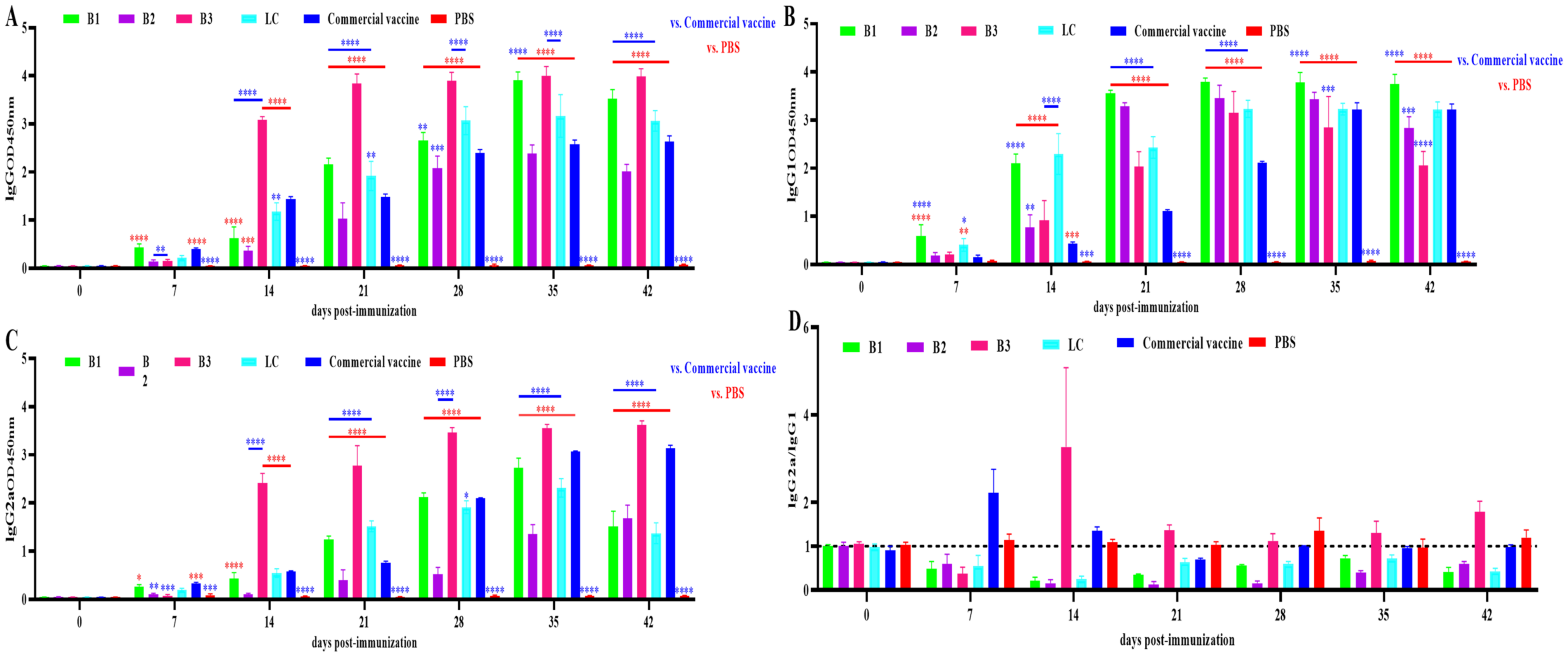
Specific antibody levels in mouse serum. The mice were immunized with in-house-prepared inactivated vaccines, with a commercial vaccine and PBS used as the PC and NC, respectively. Serum samples were collected on days 0, 7, 14, 21, 28, 35 and 42 post-immunizations. ELISA was used to quantify BVDV-specific IgG, IgG1, and IgG2a antibodies. **(A)** IgG levels in mouse serum. **(B)** IgG1 levels in mouse serum. **(C)** IgG2a levels in mouse serum. **(D)** Ratio of IgG2a to IgG1 antibodies in mouse serum. Statistical significance was assessed by two-way ANOVA, with ^*^p < 0.05, ^**^p < 0.01, ^***^p < 0.001, and ^****^p < 0.0001. Blue indicates the comparison of in-house-prepared inactivated vaccines and NC group vs. the PC group; red indicates the comparison of in-house-prepared inactivated vaccines and PC group vs. the NC group.

To determine the immunogenicity of in-house-prepared inactivated vaccines (B1, B2, B3, and LC), BALB/c mice were immunized with these vaccines. A commercial vaccine and PBS were used as the PC and NC, respectively. Serum was collected on days 0, 7, 14, 21, 28, 35 and 42 after the initial immunization, and BVDV-specific IgG, IgG1, and IgG2a levels were measured via ELISA. As shown in Figures 3A–C, the inactivated BVDV vaccine induced strong IgG, IgG1, and IgG2a responses in the mice after immunization.

The IgG2a/IgG1 ratio is used to assess the Th1/Th2 immune response induced by the vaccine, with a ratio above 1 indicating a Th1-biased response and a ratio below 1 suggesting a Th2-biased response. The ratios in mouse sera are shown in Figure 3D. In the B1, B2, and LC vaccine groups, the ratio remained below 1, suggesting a predominant Th2 response. In the B3 group, the ratio was less than 1 on day 7 (Th2) and increased above 1 between days 14 and 42 (Th1). The commercial inactivated vaccine group presented a ratio above 1 on days 7 and 14 (Th1), which then decreased to below 1 by days 21–42 (Th2).

### Determination of neutralizing antibodies

**Figure 4:**
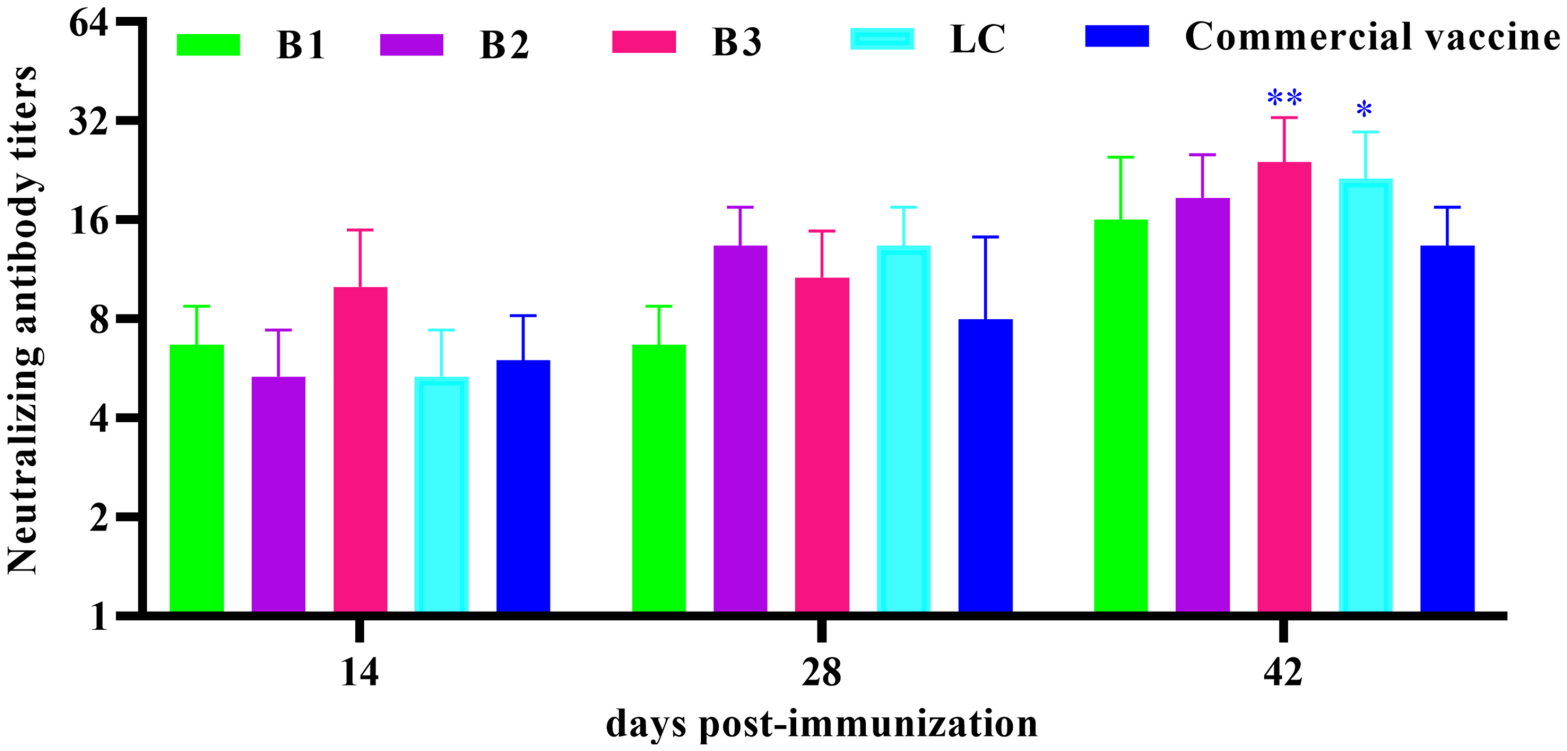
Quantification of neutralizing antibody titers in mouse serum. The mice were immunized with in-house-prepared inactivated vaccines, with a commercial vaccine used as the PC group. Serum samples were collected on days 14, 28, and 42 post-immunizations. VAT was performed to measure BVDV-neutralizing antibody titers. Statistical significance was assessed by two-way ANOVA, with ^*^p < 0.05 and ^**^p < 0.01.

The neutralizing antibody response to in-house-prepared inactivated vaccines was assessed in BALB/c mice, with a commercial vaccine used as the PC. Serum samples were collected on days 14, 28, and 42 post-immunization, and BVDV-neutralizing antibodies were quantified via the VNT. As shown in Figure 4, neutralizing antibody titers in the B3- and LC-immunized groups were significantly higher than those in the PC group on day 42 post-immunization (*p* < 0.05).

### Protection against BVDV challenge

**Figure 5:**
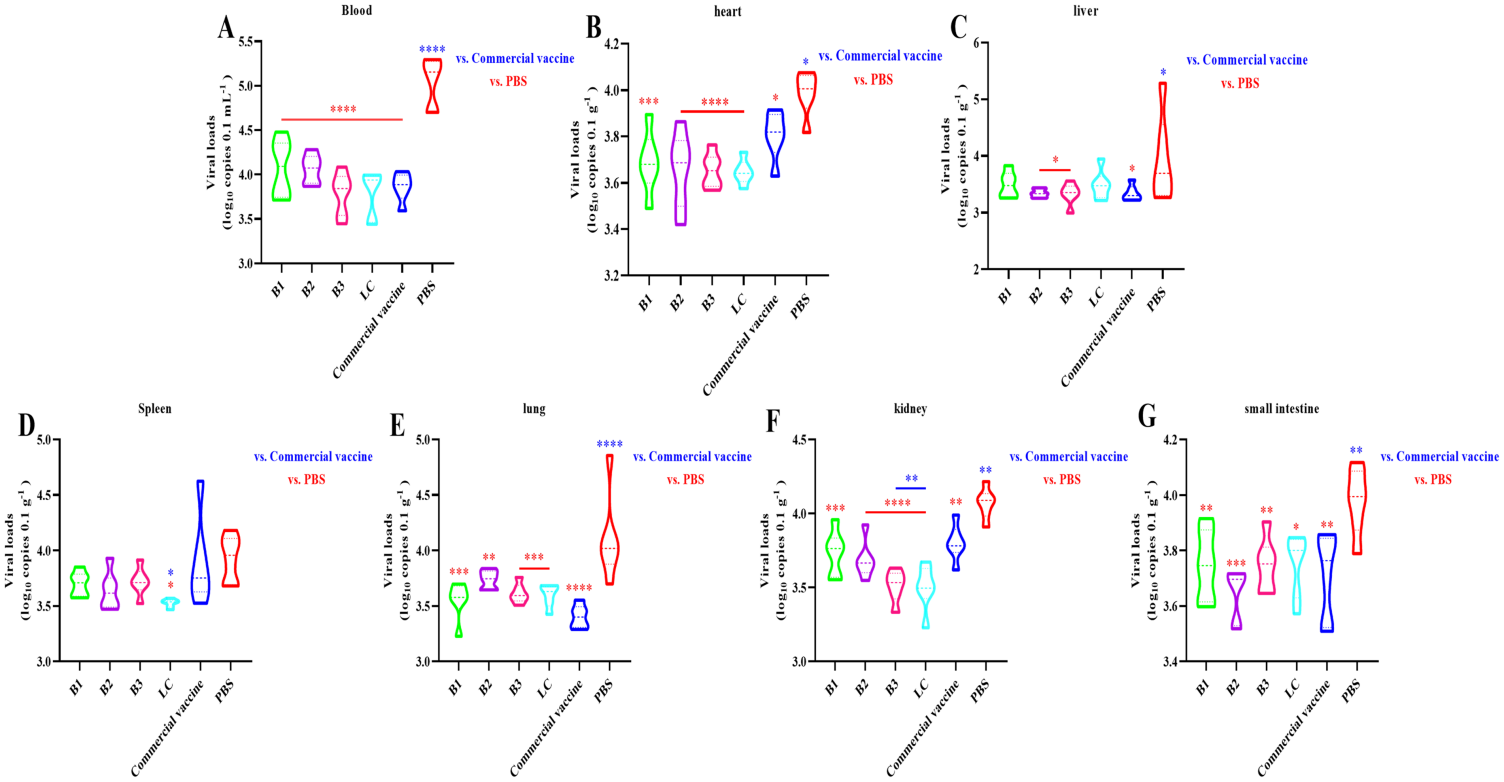
Virus loads detected by RT-qPCR. The mice were immunized with in-house-prepared inactivated vaccines, with a commercial vaccine and PBS serving as the PC and NC, respectively. Blood **(A)**, heart **(B)**, liver **(C)**, spleen **(D)**, lung **(E)**, kidney **(F)**, and small intestine **(G)** samples were collected on day 14 post-challenge. RT-qPCR was used to quantify the BVDV load. Statistical significance was assessed by one-way ANOVA, with ^*^p < 0.05, ^**^p < 0.01, ^***^p < 0.001, and ^****^p < 0.0001. Blue indicates the comparison of in-house-prepared inactivated vaccines and NC group vs. the PC group; red indicates the comparison of in-house-prepared inactivated vaccines and PC group vs. the NC group.

We established a standard curve with a strong linear relationship (*y* = −3.226*x* + 42.771, *R*^2^ = 0.999; Supplementary Figure 3) to quantify the viral loads. RT-qPCR was used to measure the viral loads in the blood, heart, liver, spleen, lung, kidney, and small intestine 14 days post-challenge. The results revealed significantly lower viral loads in the blood, heart, lung, kidney, and small intestine of the mice immunized with the B1 inactivated vaccine than in those of the NC group (Figures 5A,B,E–G; *p* < 0.01). In the B2, B3, and PC groups, the viral loads in the spleen were similar to those in the NC group, whereas the viral loads in other tissues were significantly reduced (Figures 5A–G; *p* < 0.05). Notably, the viral loads in the kidneys of the B3 group were significantly lower than those in the PC group (Figure 5F; *p* < 0.01). In the LC vaccine group, the viral loads in the liver were comparable to those in the NC group, but significant reductions were observed in the heart, lung, small intestine, and other tissues (Figures 5A–G; *p* < 0.05). Additionally, the viral loads in the spleen and kidney of the LC group were significantly lower than those in the PC group (Figures 5D,F; *p* < 0.05). These findings suggest that in-house-prepared inactivated vaccines, as well as commercial vaccines, provide superior protection compared with NC, resulting in significant reductions in viral loads across multiple tissues.

## Discussion

BVDV is a major pathogen in the global cattle industry and is responsible for substantial economic losses and reduced animal welfare (Wernike et al., 2024). Despite its high morbidity and relatively low mortality, BVDV often receives insufficient attention, contributing to its widespread transmission. The continued expansion of the cattle industry may lead to the emergence of novel BVDV genotypes or subtypes. Vaccination remains a primary strategy for preventing BVDV infection. Many countries currently use MLV vaccines, inactivated vaccines, or combination vaccines for disease control. However, inactivated vaccines approved in China are predominantly designed for BVDV genotype 1 strains, limiting their efficacy against newly emerging genotypes or subtypes. Therefore, developing vaccines targeting regional BVDV strains is essential to achieve more robust and long-lasting immunity.

In this study, four field strains of BVDV (B1, B2, B3, and LC) were isolated from the Xinjiang region and characterized. These strains were cultured and concentrated, yielding viral titers of 10^8^ TCID_50_/mL, 10^7.5^ TCID_50_/mL, 10^7.88^ TCID_50_/mL, and 10^7.67^ TCID_50_/mL, respectively. A review of commercially available inactivated BVDV vaccines indicated that the viral antigen content typically does not fall below 10^7^ TCID_50_/mL (Wu et al., 2017). Therefore, we standardized the viral antigen content in our study to 10^7.5^ TCID_50_/mL (Wang et al., 2019). To achieve effective virus inactivation, we used H_2_O_2_ as the inactivating agent, which has been shown to be both safe and effective in previous studies (Abd-Elghaffar et al., 2016; Li et al., 2024). To enhance the immune response, we incorporated Freund’s adjuvant, a widely used immunological enhancer in animal models, and mixed it with the inactivated virus at a 1:1 ratio. After emulsification, we assessed the characteristics of the resulting vaccine formulations, including their appearance, viscosity, stability, and sterility. The results revealed that all four inactivated vaccines presented a milky white color, an appropriate viscosity for injection, stability, and sterility and conformed to the relevant regulatory and quality control standards for vaccine development.

IFN-γ is the only type II interferon and pivotal immune cytokine that is primarily secreted by activated T lymphocytes and natural killer (NK) cells (Boehm et al., 1997; Schroder et al., 2004). It not only regulates T-cell differentiation and promotes Th1-type immune responses but also plays a crucial role in both cellular and humoral immunity (Smith and Denning, 2014). Furthermore, IFN-γ is involved in modulating inflammatory responses, as well as in antitumor and antiviral immunity (Boehm et al., 1997; Samuel, 2001). As such, IFN-γ is considered a key regulatory factor in the immune system. In this study, spleen lymphocytes were harvested from mice on days 28 and 42 post-immunization and stimulated with the relevant antigens to assess IFN-γ secretion levels. Compared with those from the NC group, spleen lymphocytes from the inactivated vaccine group presented a significant increase in IFN-γ secretion, with a noticeable increasing trend. Notably, the IFN-γ secretion levels in the B1 (28 days post-immunization) and B3 (42 days post-immunization) groups were significantly higher than those in the PC group, indicating that the prepared inactivated vaccine effectively induced a robust cellular immune response.

IgG is the most abundant serum antibody, with IgG1 and IgG2a being the predominant subclasses (Vidarsson et al., 2014). The ELISA results demonstrated that immunization with the inactivated vaccine induced specific IgG, IgG1, and IgG2a antibodies in the mice, confirming the capacity of the vaccine to activate the adaptive immune system. The IgG2a/IgG1 ratio serves as a key indicator of the Th1/Th2 immune response balance elicited by the vaccine (Ferreira et al., 2008). Th1 responses, which are involved in cell-mediated immunity, include macrophage activation, enhancement of cytotoxic T lymphocytes (CTLs), and IFN-γ production, all of which target intracellular pathogens (Coakley et al., 2019). In contrast, Th2 responses are associated with humoral immunity, promoting B-cell differentiation into plasma cells, leading to antibody production and the elimination of extra-cellular pathogens (Ellenbogen et al., 2018). Additionally, Th2 responses play a critical role in allergic regulation (Romagnani, 2004; Karp, 2010). In this study, the inactivated B1, B2, and LC vaccines elicited predominantly humoral immune responses. In contrast, the B3 inactivated vaccine initially induces a humoral immune response, which later transitions to a cellular immune response. Commercially available inactivated vaccines primarily trigger a cellular immune response early, followed by a shift to a humoral immune response. Neutralizing antibodies, which block viral entry into host cells, are crucial for preventing viral infections (Cromer et al., 2021). The virus neutralization assay remains the gold standard for the serological diagnosis of BVDV and is a key measure of vaccine efficacy (Chimeno Zoth et al., 2007; Yi et al., 2022). Our data indicate that the inactivated vaccine induced neutralizing antibodies in mice, with titers progressively increasing over time. Notably, 42 days post-immunization, the neutralizing antibody titers in the B3 and LC groups were significantly higher than those in the PC group (Figure 4; *p* < 0.01). Challenge experiments revealed that 42 days after immunization, the B3 group presented significantly lower viral loads in multiple tissues than did the PC group (Figures 5D,F; *p* < 0.05). These findings suggest that the prepared inactivated vaccine effectively reduced the viral load and provided substantial protection against BVDV infection. In conclusion, the B3 strain effectively induce both humoral and cellular immune responses, showing considerable promise for the development of commercially viable inactivated vaccines. These findings provide strong evidence for the control and eradication of BVDV in the Xinjiang region.

The mouse model is extensively used in preclinical research, particularly in the development of novel vaccines and therapeutics. While infection with BVDV in mice typically does not produce overt clinical symptoms, studies have demonstrated that BALB/c mice, when BVDV is administered via intraperitoneal injection, exhibit characteristic clinical manifestations and significant pathological tissue damage (Seong et al., 2015; Quintana et al., 2020; Liu et al., 2021). This has confirmed the susceptibility of mice to BVDV, establishing the mouse model as a widely accepted tool for BVDV vaccine development (Yang et al., 2022; Li et al., 2024). Given its advantages, including ease of handling, low cost, rapid data generation, and manageable animal care requirements, we selected BALB/c mice as the experimental model for this study to evaluate the immunogenicity of the inactivated vaccine formulation.

However, despite its utility in generating preliminary immunological data for BVDV vaccine evaluation, the mouse model has inherent limitations. Notably, significant differences in immune system characteristics between mice and cattle, such as T cell subset distribution and cytokine responses, may hinder its ability to fully replicate the immune response in cattle (Vlasova and Saif, 2021; Gómez-Romero et al., 2023). Consequently, this discrepancy could impact the accuracy of vaccine efficacy predictions. While the mouse model remains valuable for initial vaccine screening, subsequent studies will prioritize further validation of the immune response in cattle models to more accurately assess the clinical potential of BVDV vaccines.

## Conclusion

In this study, H_2_O_2_ was used to inactivate the B1, B2, B3, and LC strains isolated from Xinjiang, and inactivated vaccines were developed specifically for these regionally prevalent strains. After immunizing mice, the B3 inactivated vaccine induced strong cellular and humoral immune responses, significantly enhancing γ-interferon production, specific antibodies, and neutralizing antibodies, while effectively inhibiting viral replication. Comprehensive evaluation revealed that the B3 vaccine exhibited superior immunogenicity compared to commercially available vaccines, highlighting its potential for future development. However, since this study was conducted using a mouse model, clinical trials in target animals, such as cattle, are needed for further validation. Furthermore, as the vaccine progresses from laboratory research to large-scale production, challenges related to production scale, quality control, cost-effectiveness, and regulatory compliance must be addressed to ensure its safety and efficacy in real-world applications.

## Data Availability

The original contributions presented in the study are included in the article/Supplementary material, further inquiries can be directed to the corresponding authors.
