# Supplementary figures and images for: Inactivated vaccines derived from bovine viral diarrhea virus B3 strain elicit robust and specific humoral and cellular immune responses
Source: Front Microbiol. 2025 Aug 8;16:1607334. doi: 10.3389/fmicb.2025.1607334 (PMC12371705; doi:10.3389/fmicb.2025.1607334)

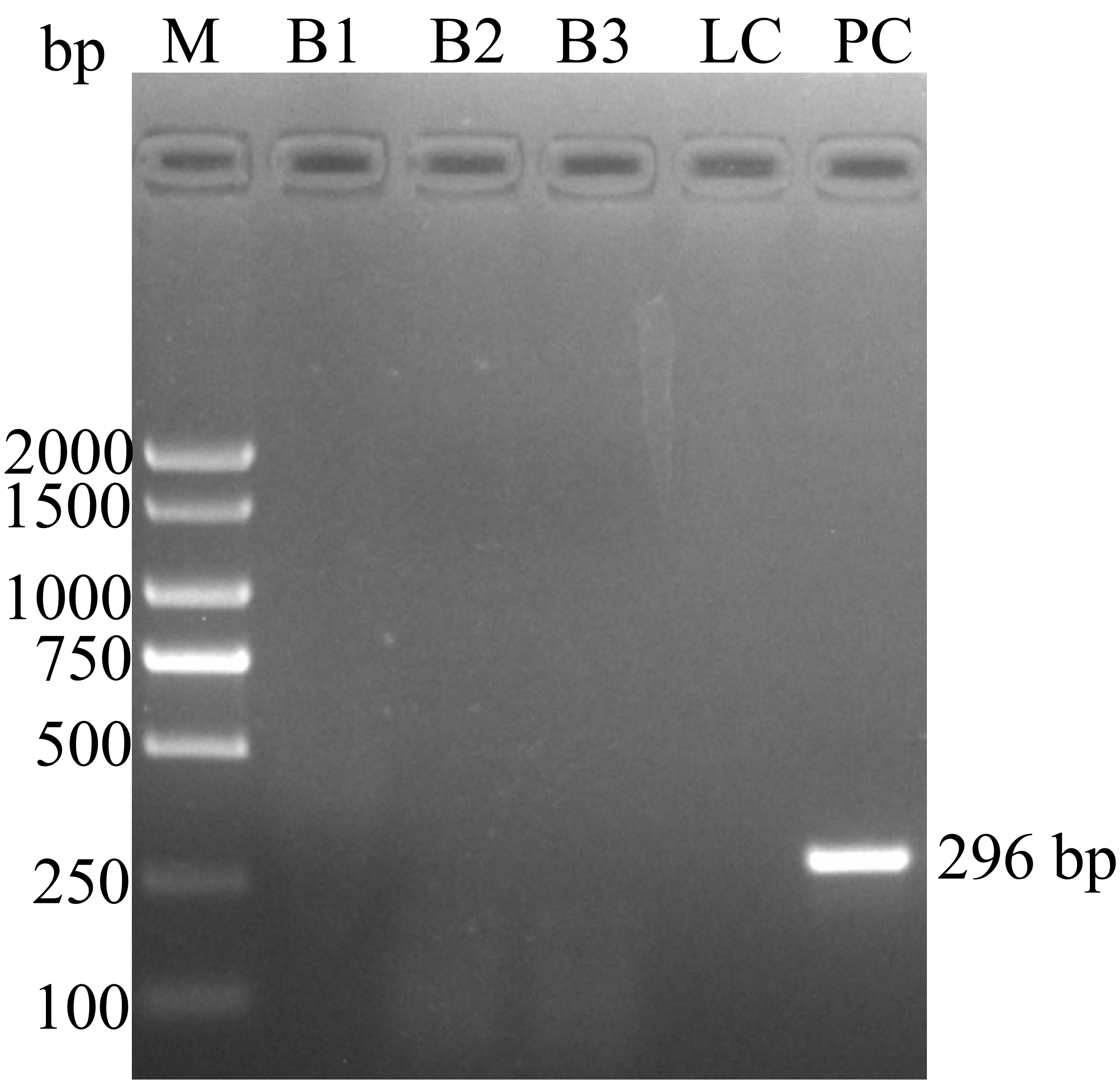

Supplement: Supplementary file 1 [file Image_1.tif]

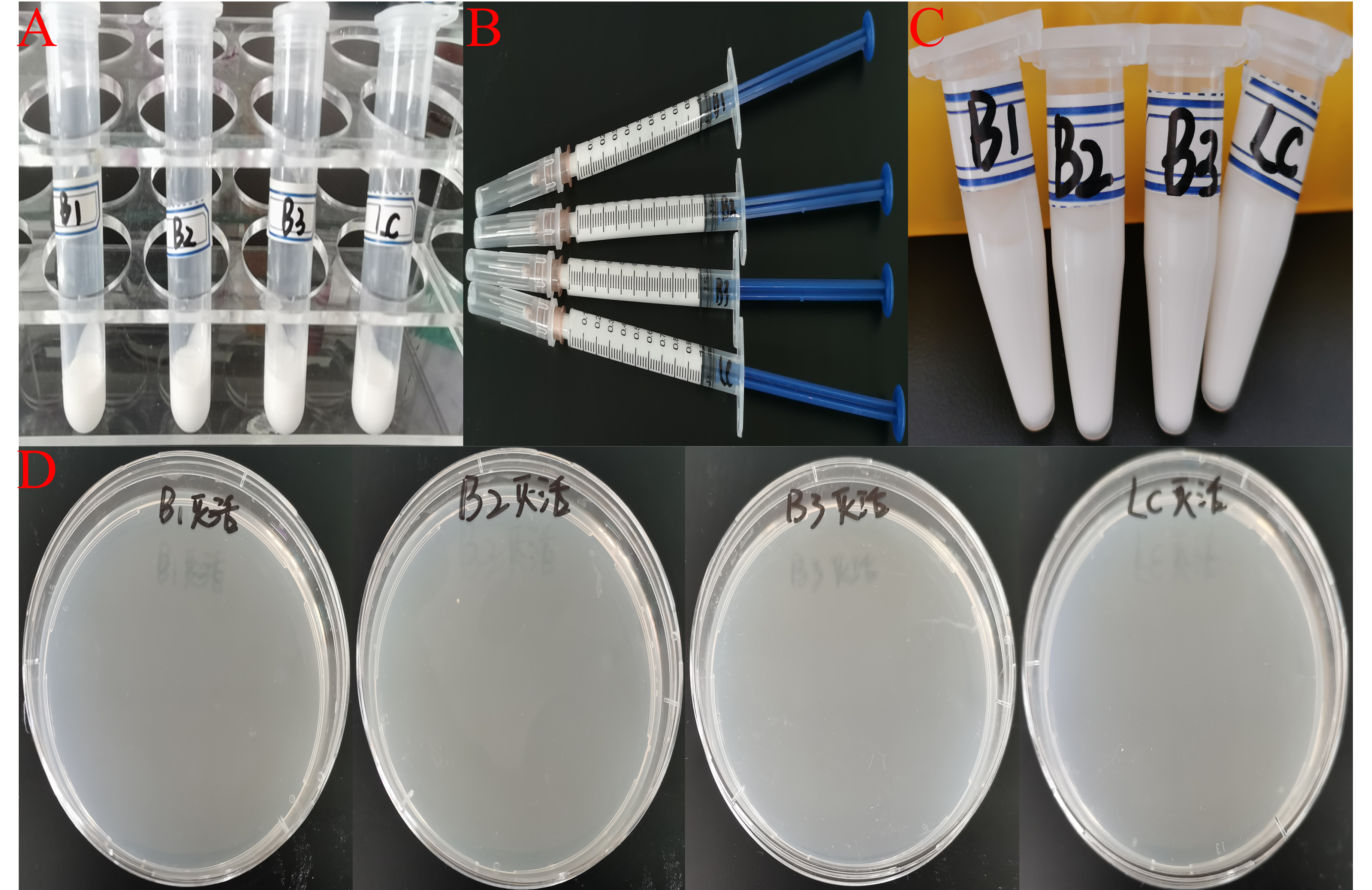

Supplement: Supplementary file 2 [file Image_2.tif]

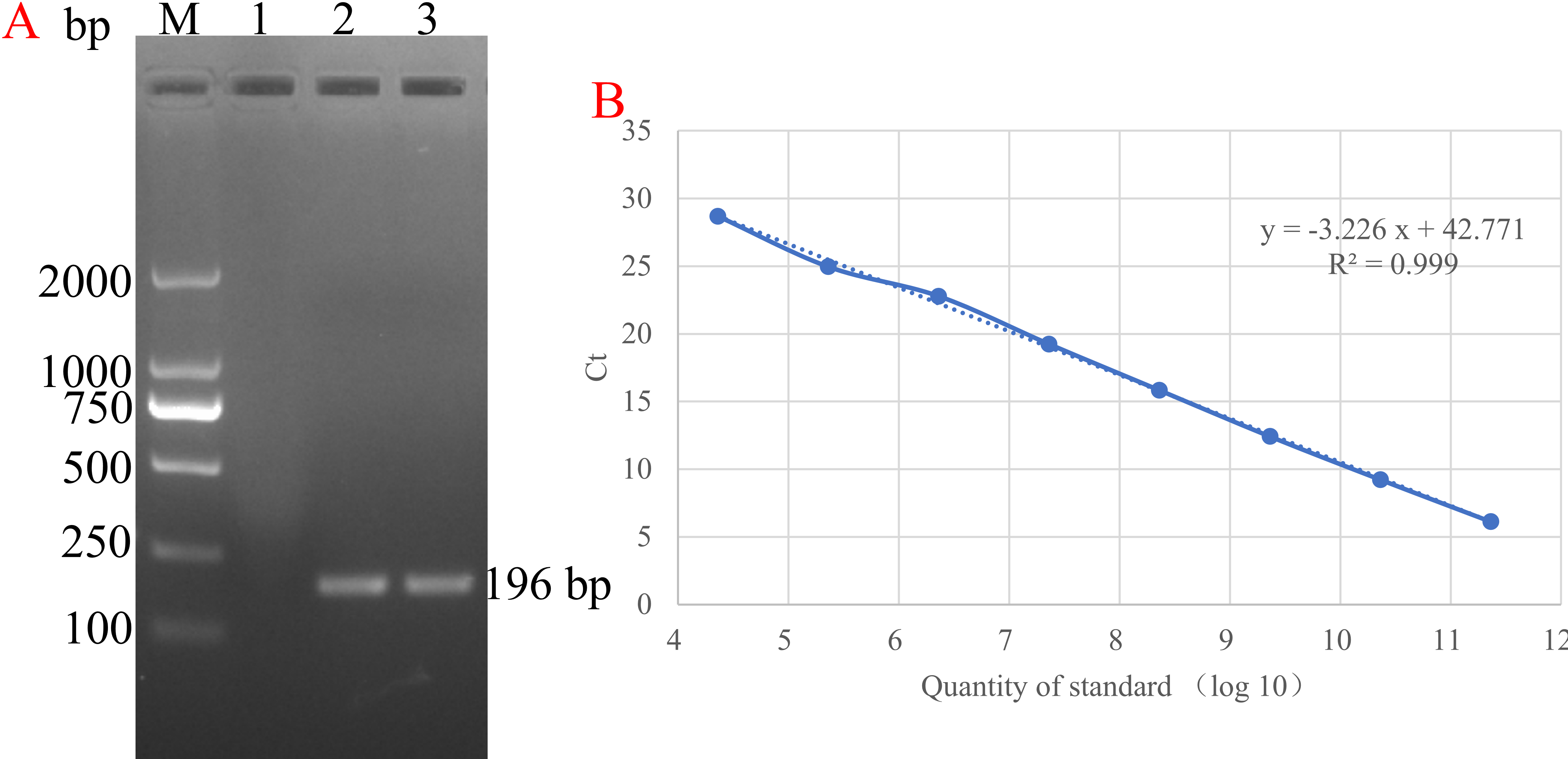

Supplement: Supplementary file 3 [file Image_3.tif]
